# Reimagining care for young adults living with type 1 diabetes

**DOI:** 10.1111/jdi.13824

**Published:** 2022-05-16

**Authors:** Eimear C Morrissey, Sean F Dinneen, Michelle Lowry, Eelco JP de Koning, Marleen Kunneman

**Affiliations:** ^1^ Health Behavior Change Research Group School of Psychology National University of Ireland Galway Ireland; ^2^ School of Medicine National University of Ireland Galway Ireland; ^3^ Centre for Diabetes, Endocrinology and Metabolism Galway University Hospitals Galway Ireland; ^4^ Department of Medicine Leiden University Medical Center Leiden the Netherlands; ^5^ Medical Decision Making, Department of Biomedical Data Sciences Leiden University Medical Center Leiden the Netherlands; ^6^ Knowledge and Evaluation Research Unit Mayo Clinic Rochester Minnesota USA

**Keywords:** Delivery of healthcare, Diabetes mellitus, type 1, Young adult

## Abstract

Young adults living with type 1 diabetes often struggle to achieve what clinicians consider to be optimal levels of metabolic control. Despite the impact that this can have on a young person's future risk of complications, there are relatively few studies reporting new ways of organizing or delivering care to this cohort. In this article, we explore some of the reasons why young adult diabetes care is challenging, and describe approaches to “re‐imagining” how care might be improved. The work is informed by the ‘Making Care Fit’ collaborative and by a program of research, entitled D1 Now, involving co‐design of a complex person‐centered intervention with young adults.

## Graphical abstract

Young adults living with type 1 diabetes often struggle to achieve what clinicians consider to be optimal levels of metabolic control. Despite the impact that this can have on a young person’s future risk of complications, there are relatively few studies reporting new ways of organizing or delivering care to this cohort. In this paper we explore some of the reasons why young adult diabetes care is challenging and describe approaches to “re‐imagining” how care might be improved.

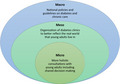

## INTRODUCTION

Living with type 1 diabetes is a challenge at any stage of the life course, but especially in young adulthood. This phase of human development is often characterized by the young person, typically aged 18–25 years, moving away from the family home to independent living, as they take on higher education or work responsibilities. It is often associated with ‘risk taking’ behavior, as the young person explores opportunities that might not have been available to them previously, including use of alcohol or recreational drugs. Developing one's own identity (separate from that imposed by parental expectations) and establishing one's own path in life can be stressful, particularly if issues of body image, gender identity and/or sexual orientation are also at play. In the modern world, where social media can be both a help and a hindrance, the pressures associated with young adulthood seem to be increasing rather than decreasing.

Alongside navigating these challenges associated with young adulthood, a person living with type 1 diabetes is also expected to take on additional responsibility in the day‐to‐day self‐management of their diabetes. Transition from pediatric to adult diabetes services typically happens at approximately 18 years of age. Depending on the health service in which the young person is receiving their diabetes care, the process of transition can run smoothly or be associated with additional frustrations. The well‐established working relationship that often exists between pediatric teams and the young person (and their family) suddenly ends, and a whole new team of clinicians appear. Given these many life course and health system challenges, it is not surprising that many young people living with type 1 diabetes struggle with achieving optimal metabolic control^1^.

In the present article. we have tried to ‘re‐imagine’ how diabetes care might be delivered to young adults. The article has resulted from a collaboration between diabetes caregivers and researchers based in Ireland and in the Netherlands. Some of the shared beliefs that have led to us writing this paper include:

• the young person's biography (by which we mean their life experiences and those of their loved ones, their expectations and priorities) is as important as their biology (by which we mean certain metabolic parameters that are easy to measure before or during a clinic visit);
• clinic non‐attendance and high levels of glycated hemoglobin (HbA1c) are as much, if not more, a reflection of failure on the part of the health system delivering care as they are on the part of the young person receiving care;
• although we might aspire to deliver person‐centered care, often the systems we design for care delivery are geared more toward efficiency of practice (care for ‘people like this’), and not the expressed needs and wishes of the young person (care for ‘this person’);
• including the voice of the young person in service design (or re‐design) is a key element to improving outcomes of care.

**Figure 1:**
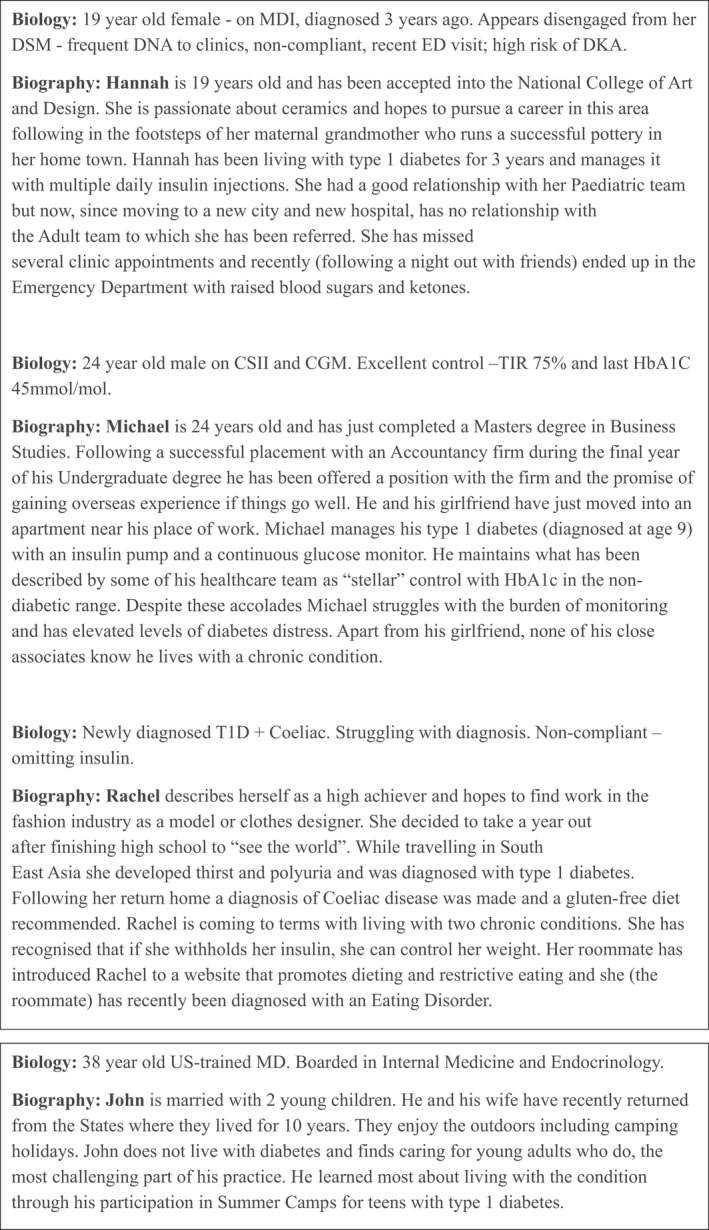
Vignettes. CGM, continuous glucose monitoring; CSII, continuous subcutaneous insulin infusion; DKA, diabetic ketoacidosis; DNA, did not attend; DSM, diabetes self‐management; ED, emergency department; HbA1c, glycated hemoglobin; MDI, multiple daily doses of insulin; T1D, type 1 diabetes.

In writing this article, we draw heavily on a program of young adult‐focused research in Ireland, entitled D1 Now, and on the Making Care Fit collaborative coordinated from the Netherlands^2^. We begin with a series of vignettes (Figure 1) intended to show some of the people who participate in young adult clinic activity. The descriptions draw on the Language Matters literature^3^, and attempt to show the reality (biology) and the ideal (biography) of how we communicate in the clinical environment.

## IMPORTANCE OF CO‐DESIGN

Designing care that fits for young adults living with type 1 diabetes requires these young adults, their loved ones and their clinicians to work together. As evidenced by the vignettes in Figure 1, the clinician often only sees a biomedical snapshot of what is happening in a young person's life. To co‐create a plan of work that makes sense, the clinician needs to understand the situation of the patient, beyond the biology. This work of making care fit takes place mostly during clinical encounters at the point of care. As young adults implement the plans in their personal environment, they work to make care fit at the point of life. The young adult is usually the one person to fully bridge these two worlds, and whatever is left undiscussed with their clinician at the point of care, is also left unconsidered when designing care plans. This in turn leads to care plans that these young adults do not need, understand or cannot implement at that point of life. This result is both wasteful and potentially harmful^2^, ^4^.

Previous work has identified that some young adults feel they do not always have a collaborative relationship with their clinicians^5^, which means that care plans are unlikely to fit in with their lives. The D1 Now study was set up with the aim of bridging this gap and reimagining how care is delivered to young adults. Recognizing that co‐design is a crucial component to success, a Young Adult Panel of 10 young adults living with type 1 diabetes was established^6^. These young adults act as co‐researchers in the work. The D1 Now intervention has been designed through rounds of qualitative research with young adults and diabetes staff, with input from the Young Adult Panel at each stage^7^, ^8^.

## SUPPORTING YOUNG ADULTS' PRIORITIES AND MINIMALLY DISRUPTING THEIR LIVES

To make care fit, we need to maximally support young adults' priorities. This tends not to happen in current practice, where there is a tendency for clinical encounters to focus on HbA1c above all else. This narrow focus might fail to consider multimorbidity (see Rachel's vignette), as well as values, preferences, needs, wishes, and financial, family and social facets of the young adult's life^2^, ^4^. It also fails to take mental health into account; Jones, Vallis and Pouwer argue that the pursuit of psychological well‐being is as important as physical health for people living with diabetes^9^.

People living with diabetes have highlighted their dissatisfaction with the sole focus on HbA1c. Participants in the MILES‐2 study, which surveyed >1,000 people living with diabetes in Australia (54% living with type 1 diabetes), felt their clinicians’ focus was too narrow and biomedical^10^, and young adults in the DiaPROM study reported that they were reluctant to attend the clinic when the focus was only on the (biomedical) numbers^11^. We need to shift the focus of clinical encounters from the narrow biomedical focus to one that is inclusive of young adults' own priorities and life context.

In the D1 Now study, two of the intervention components, the Support Worker and Agenda Setting Tool, were designed and developed to improve the young adult's clinical encounter, and support young adults and clinicians to co‐create a plan that fits at the point of life. They work by shifting the focus of the encounter from the biomedical to the wider medical and personal contexts. The support worker aims to develop a collaborative and holistic relationship with the young adult, and advocate for their needs in the clinic^12^. The agenda setting tool is intended to promote a shared decision‐making approach to the clinic consultation; it includes sections that are filled in by the young adult before their consultation, and sections that are filled in jointly with their clinician during the consultation. The first question asks, “What do you want to talk about at your clinic appointment today?”. When trialed during the D1 Now pilot randomized controlled trial, this question was sometimes answered with ‘HbA1c’, but more frequently answered with requests for new diabetes technology or education, considerations of other comorbid health conditions, and questions around exercise, motivation, college life, travel, alcohol, career changes, hypos, stress, support groups and driving licenses. Experience with introducing the agenda setting tool into routine clinical practice has highlighted the importance of providing training for clinicians in its use^13^. These intervention components serve as two examples of how aspects of care can be co‐designed to enhance the clinical encounter and allow all parties to work together to create a plan that fits at that point of life.

When designing care plans that are most effective and pursue young adults' priorities for their life, health and care, we must recognize that people have a finite, and varying, capacity to prevent disruption, to cope and to adapt^2^. Higher workload delegated to young adults and their social networks, sometimes called ‘treatment burden’, leads to reductions in quality of life^14^. This is particularly pertinent to young adults with type 1 diabetes, as they are expected to implement complicated treatment regimens in which they continuously estimate and administer insulin doses, monitor glucose levels, and so on. Importantly, a recent exploration showed that three of every four young adults with type 1 diabetes indicate that their investments of time, energy and efforts in healthcare are unsustainable over time^15^. The workload of healthcare might be tolerated better when ‘the work of being a patient’ is more clearly connected to advancing people's goals and priorities, and when efforts are put in place to minimally disrupt their lives (‘minimally disruptive medicine’^16^). Not only must the demands of healthcare be feasible within the life of young adults and their social networks, but healthcare should also minimally disrupt those aspects of their lives they treasure the most, and which justify the pursuit of care in the first place. For care to fit, we must understand how each person (and not ‘people like this person’) lives their life, and how life is aimed to be lived^2^, ^4^.

The need to align diabetes treatment with patient goals is recognized in the latest guidelines by the European Association for the Study of Diabetes and the American Diabetes Association, by recommending flexible treatment goals and programs, and by recognizing burden of treatment as a key consideration. Shared decision‐making tools are useful aids in identifying and acting on patients' priorities. These tools guide patients and clinicians through the process of shared decision‐making; fostering of choice awareness, discussing the available options, exploring patient priorities and making a final decision^17^. Several initiatives are in place to improve personal goal setting of people with diabetes. One such initiative is the Instrument for Patient Capacity Assessment (I‐CAN) conversation tool for use within the clinical encounter. This tool helps patients and clinicians to discuss if and how care plans might interact with the patient's life, and in discovering how aspects of the patient's life might interact with the care plan^18^.

## YOUNG ADULT AND CLINICIAN COLLABORATION

These efforts of making care fit require young adults (and their loved ones) and clinicians to collaborate. In the context of young adult diabetes care, and indeed perhaps in healthcare more broadly true (and humanistic) patient‐clinician collaboration is often not realized^19^. When thinking about patient‐clinician collaboration, it is important to acknowledge the context within which the relationship has been established. The clinician typically enters this relationship with a high degree of choice and power, financially rewarded for their contributions in an area they have elected to pursue professionally and can switch off once they leave the clinic environment. Clinicians also play a gatekeeper role in terms of their power to grant or deny access to healthcare support, technologies and medicines for patients. The young adult, in contrast, enters this relationship not by choice, but by necessity, having received a diagnosis they did not ask for, but are now living with. In a life stage often characterized by independence, young adults often face a struggle due to the relative reliance they have on the clinician to access the healthcare services and supports they need. Combining the professional knowledge of the clinician and the lived experience knowledge of the person with type 1 diabetes can (if done well) lead to a win–win situation, where expert patients interact with person‐centered clinicians to achieve optimal outcomes that meet the needs of the person receiving care.

The power dynamic that is often present in the consultation has been challenged recently by events, both planned (e.g., advances in diabetes technology and peer support groups) and unplanned (e.g., the coronavirus disease 2019 pandemic). Who would have thought 10 years ago that a Do‐It‐Yourself Artificial Pancreas System movement of patients and parents not willing to wait for MedTech companies to disseminate hybrid closed loop technology would take it on themselves to do so^20^, ^21^? Who would have thought that video consultations and remote diabetes education delivered in patients' homes would become the norm, effectively reducing the gap between the point of care and the point of life? Who would have thought that the diabetes online community would mobilize so strongly, and have people living with diabetes influencing and challenging the agendas of international diabetes conferences^22^? The next generation of clinicians will need to embrace these changes in practice, and become more responsive to the ever‐changing needs of the young person living with diabetes.

Training programs for clinicians should embrace opportunities to gain a better understanding of biography as well as biology. Counseling and empowerment skills training courses can help clinicians recognize that only the person living with diabetes can change their behavior, thereby helping the clinician move from ‘fixer’ to ‘facilitator’. Like John, the doctor in our vignette, efforts to understand what it is really like to live with type 1 diabetes could be the strongest part of the professional diabetes learning journey.

## MAPPING OUR EVALUATION OF PROGRESS

In realizing our re‐imagined healthcare, how and when do we evaluate our progress? Above, we have made the case that we need to look beyond HbA1c levels or other biomedical markers. Signals of care that fail to fit in young adults' lives might come from ‘uncontrolled’ HbA1c levels, or ‘non‐compliance’, but we often remain blind to those young adults whose type 1 diabetes seems well‐controlled, but only at the cost of those aspects of life that make life worth living in the first place (see Michael's vignette).

Evaluating efforts of making care fit, both at the point of life and at the point of care, should include evaluating whether care maximally supports and accounts for the young adults' situation and priorities, and minimally disrupts their lives, loved ones and social networks. It should include evaluating both the content and the manner of collaboration between young adults and their clinicians. Finally, it should include evaluating the ongoing style of care; continuously re‐evaluating whether care still fits in people's lives, and people's lives can still be lived in their care plans^2^. In doing so, we might need to prioritize evaluation of ‘this person’ over evaluation of ‘people like this person’^2^, focusing on outcomes that matter in care and in life, and on the rational, emotional and practical sense of care plans to young adults^15^. Relevant outcomes might differ between people and even within one person between timepoints. Although such evaluation is, in some way, quantifiable, it is likely that words will also be required to help interpret these numbers. As Iona Heath stated, the need to involve human judgment in making meaning of descriptions “is perhaps the one attribute of words that make them so peculiarly appropriate for judging quality within healthcare”^23^. All this might help us avoid what we previously called ‘measurement with a wink’; focusing on acing the test and getting high scores on measures to ‘provide the illusion of good, better or improved care, while favoring measurable care that is predominantly standard, technical, mechanical and context‐blind’^24^.

## CONCLUDING THOUGHTS

**Figure 2:**
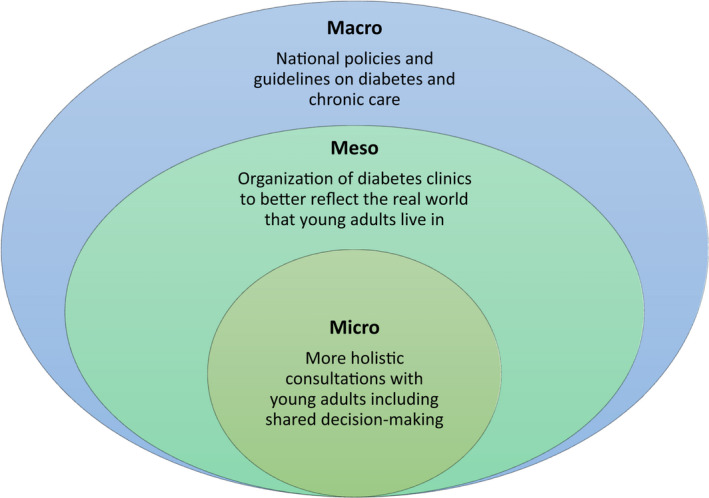
Re‐imagining young adult diabetes care will require changes to occur at several different levels in the health system to facilitate a more patient‐centered approach. [Colour figure can be viewed at wileyonlinelibrary.com]

The journey that we are on of trying to re‐imagine care for young adults living with type 1 diabetes has highlighted how challenging it can be to effect lasting change in the way we organize and deliver care. We remain convinced that this change is necessary, and we believe it will require addressing issues at the micro, meso and macro level^23^ (Figure 2).

At the micro level, reflected in the individual consultation, we need to help clinicians understand the biography, as well as the biology, of their patient. This can be facilitated through training in person‐centered consultations and use of shared decision‐making or conversation tools that help both the clinician and patient to devise care plans that make sense. We need to facilitate patients expressing their concerns and frustrations with the burden of self‐management. This requires a more holistic view from the clinician of what diabetes care is all about.

At the meso level, reflected in the way that clinics and clinical encounters are organized, we need to recognize that the hospital outpatient facility is not the real world. The point of life, as opposed to the point of care, is where real impact occurs. Clinicians need to be willing to explore and better understand the world where young people live and where care plans are (or are not) implemented. Home visits occur in geriatric medicine, but would be unheard of in adolescent or young adult care. Yet, the COVID‐19 pandemic has taught us that video consultations have a place in care delivery, and can help bridge the gap between the point of care and the point of life.

At the macro level, reflected in national policies and guidelines, we need to reward organizations that are willing to move away from a system of reimbursement that is based only on HbA1c targets or other measures of ‘people like this person’. Identifying and measuring outcomes that are more important to young people living with type 1 diabetes and rewarding systems that promote a more holistic approach (including assessing the burden of treatment) should be the goal. The patient’s voice needs to be incorporated at all levels to ensure that any change is effective.

## DISCLOSURE

The authors declare no conflict of interest.

Approval of the research protocol: N/A.

Informed consent: N/A.

Registry & registration no. of the study/trial: N/A.

Animal studies: N/A.
